# Gene expression identifies heterogeneity of metastatic behavior among gastrointestinal stromal tumors

**DOI:** 10.1186/s12967-016-0802-3

**Published:** 2016-02-13

**Authors:** Keith M. Skubitz, Kate Geschwind, Wayne W. Xu, Joseph S. Koopmeiners, Amy P. N. Skubitz

**Affiliations:** Department of Medicine, The University of Minnesota Medical School, Minneapolis, MN USA; Masonic Cancer Center, The University of Minnesota Medical School, Minneapolis, MN USA; Department of Biochemistry and Medical Genetics, Faculty of Medicine, University of Manitoba, The Research Institute of Oncology and Hematology, Cancer Care, Winnipeg, MA Canada; Division of Biostatistics, University of Minnesota School of Public Health, Minneapolis, USA; Department of Laboratory Medicine and Pathology, University of Minnesota Medical School, Minneapolis, MN USA

**Keywords:** Microarray, Sarcoma, GIST, Gene expression, Heterogeneity, Subgroups, Metastasis, Prognosis

## Abstract

**Background:**

Adjuvant imatinib is useful in patients with gastrointestinal stromal tumors (GIST) at high risk of recurrence. At present, the risk of recurrence is determined based on tumor size, mitotic rate, tumor site, and tumor rupture. Previous studies using various biochemical pathways identified gene expression patterns that distinguish two subsets of aggressive fibromatosis (AF), serous ovarian carcinoma (OVCA), and clear cell renal cell carcinoma (RCC). These gene sets separated soft tissue sarcomas into two groups with different probabilities of developing metastatic disease. The present study used these gene sets to identify GIST subgroups with different probabilities of developing metastatic disease.

**Methods:**

We utilized these three gene sets, hierarchical clustering, and Kaplan–Meier analysis, to examine 60 primary resected GIST samples using Agilent chip expression profiling.

**Results:**

Hierarchical clustering using both the combined and individual AF-, OVCA-, and RCC- gene sets identified differences in probabilities of developing metastatic disease between the clusters defined by the first branch point of the clustering dendrograms (p = 0.029 for the combined gene set, p = 0.003 for the AF-gene set, p < 0.001 for the OVCA-gene set, and p = 0.003 for the RCC-gene set).

**Conclusions:**

Hierarchical clustering using these gene sets identified at least two subsets of GIST with distinct clinical behavior and risk of metastatic disease. The use of gene expression analysis along with other known prognostic factors may better predict the long-term outcome following surgery, and thus restrict the use of adjuvant therapy to high-risk GIST, and reduce heterogeneity among groups in clinical trials of new drugs.

**Electronic supplementary material:**

The online version of this article (doi:10.1186/s12967-016-0802-3) contains supplementary material, which is available to authorized users.

## Background

Gastrointestinal stromal tumors (GISTs) are the most common sarcoma of the gastrointestinal tract, occurring mostly in the muscular wall of the stomach or small bowel, where it is felt to arise from the interstitial cells of Cajal or similar cells [1, 2]. The primary treatment for GIST is surgical excision, but a significant number of cases recur [3, 4]. Adjuvant imatinib, a tyrosine kinase inhibitor, is useful in select cases of GIST based on risk of recurrence [5–8]. At present, the risk of recurrence is determined based on tumor size, mitotic rate, tumor site, and tumor rupture [1, 5, 8–13], as for example in the Miettinen risk score [11], but more accurate predictors would be useful to better direct therapy.

While most GISTs have mutations in the KIT gene, mutations in the platelet derived growth factor receptor alpha (PDGFRA) gene are also common [1, 2, 5, 8, 14]. In a small percentage of GISTs, mutations in other genes such as BRAF, succinate dehydrogenase (SDH), or neurofibromatosis (NF) may occur [1, 5, 15–20]. The type of KIT or PDGFRA mutation may affect the recurrence rate as well as response to imatinib [5, 8]. Despite the key role of activating mutations of KIT or PDGFRA, GIST biology is also dependent upon other genetic changes [1]. Cases of KIT-mutant GIST have been reported that present with coexisting downstream mutations [5, 8, 21, 22].

Gene expression patterns have been used to predict the development of metastases in soft tissue sarcoma [23–26]. Differences in the gene expression profiles of GISTs with different KIT- or PDGFRA-mutant tumors have been reported [27, 28], and several recent studies have explored the use of gene expression patterns to predict recurrence rate of GIST [29–35].

In previously published studies using various biochemical pathways, we derived gene expression profiles that identified two subgroups of aggressive fibromatosis (AF-gene set), ovarian carcinomas (OVCA-gene set), and clear cell renal cell carcinomas (RCC-gene set) [36–39]. We previously used a gene set derived from these three studies to separate 73 high grade soft tissue sarcoma into 2 or 4 groups with different propensities of metastasis [25]. In an independent study, these gene sets were used to separate 309 high-grade soft tissue sarcoma into 2 or 4 groups with different propensity of metastasis [26].

In the present study, we utilized our three gene sets to examine a group of 60 GISTs using Agilent chip based expression profiling [33]. These gene sets successfully separated the GIST samples into subsets with different probabilities of developing disease recurrence, and may be useful to better predict who would benefit from adjuvant imatinib.

## Methods

### Samples

Sixty primary tumor samples were obtained from patients who had surgical resection of a GIST, and patients were followed without treatment until tumors recurred as previously described [33]. Frozen samples from resected primary GISTs untreated until tumor recurrence were selected from the European GIST database CONTICAGIST (http://www.conticagist.org). According to French law at the time of the study, experiments were performed in agreement with the Bioethics Law 2004 800 and the Ethics Charter from the National Institute of Cancer; all subjects signed a non-opposition statement for research use of their sample. Total RNA was extracted from each frozen tumor sample, and analyzed on Agilent Whole human 44K Genome Oligo Array (Agilent Technologies) as previously described [33]. Patient characteristics were previously described [33]. These data were kindly provided by Dr. F. Chibon, Institute Bergonie, Bordeaux, France.

### Gene sets

Three different previously described gene sets with limited overlap were used: the AF-gene set, OVCA-gene set, and RCC-gene set. These gene sets consist of 161, 173, and 138 known genes respectively [36–39]. The AF-gene set and RCC-gene set distinguished between two subgroups of AF samples and RCC samples, respectively. The OVCA-gene set distinguished borderline from invasive serous OVCA. These three gene sets were pooled resulting in a combined gene set.

### Hierarchical clustering and fold-change analysis

The AF-, OVCA-, and RCC-gene sets were used individually or combined, to cluster the 60 primary GIST samples. For clustering, genes were median centered, normalized, and then clustered by complete hierarchical clustering using uncentered correlation with Eisen clustering software [40] and viewed using the TreeView software (http://www.rana.lbl.gov) [41].

### Analysis of time to metastasis

For each data set, we used the Kaplan–Meier (K-M) method to calculate metastasis-free survival probabilities, and cumulative probabilities of metastasis (one minus survival probabilities) at critical time points (2, 4, 6, and 8 years). p values were calculated by using the log-rank test for comparing different groups. p values ≤0.05 were considered statistically significant. Analyses were performed in R version 3.0.1 [42].

## Results

### Analysis of GIST samples using the individual AF-, OVCA-, and RCC-gene sets

**Fig. 1:**
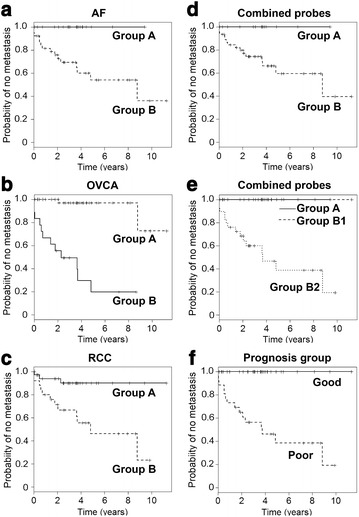
Kaplan–Meier analysis of the time to development of metastases of two groups (*groups A* and *B*) defined by the first break point of the hierarchical clustering of 60 GIST samples. GIST samples were analyzed using the individual AF-gene set (**a**), OVCA-gene set (**b**), RCC-gene set (**c**), and the combined gene set (**d**). The time to development of metastasis differed between *groups A* and *B* (**a**, p = 0.003; **b**, p < 0.001; **c**, p = 0.003; and **d**, p = 0.029). GIST samples were also analyzed by separating the samples into 3 well-separated clusters using the combined gene set (**e**, p < 0.001), and by identifying samples as “good” or “poor” prognosis by any 2 of the 3 gene sets as described in the text (**f**, p < 0.001)

We analyzed 60 GIST samples with the individual AF-, RCC-, and OVCA-probe sets; patient characteristics have been previously reported [23]. Hierarchical clustering of the GIST samples using each individual gene set (Additional file 1: Figure S1) identified differences in time to metastasis when the GIST samples were analyzed as two groups defined by the first branch point of the clustering. For the AF-gene set, the probability of not developing metastases by 6 years in Group B was 0.54 while none of the patients in Group A recurred (Fig. 1a; Table 1A, p = 0.003). For the OVCA-gene set, the probability of not developing metastases at 6 years was 0.20 in Group B vs 0.97 in group A (Fig. 1b; Table 1B, p < 0.001). For the RCC-gene set, the probability of not developing metastases at 6 years was 0.46 for Group B and 0.90 for Group A (Fig. 1c; Table 1C, p = 0.003).

**Table 1:** Probability (95 % CI) of no metastasis as a function of time

Time (years)	2	4	6	8	HR
A. Two sample subsets defined by the AF-gene set in Fig. 1a (p = 0.003)					
Group A, n = 21	NR	NR	NR	NR	Reference group
Group B, n = 39	0.76 (0.63–0.91)	0.60 (0.45–0.81)	0.54 (0.38–0.78)	0.54 (0.38–0.78)	NE
B. Two sample subsets defined by the OVCA-gene set in Fig. 1b (p < 0.001)					
Group A, n = 18	NR	0.97 (0.91–1.00)	0.97 (0.91–1.00)	0.97 (0.91–1.00)	Reference group
Group B, n = 42	0.53 (0.34–0.81)	0.28 (0.12–0.67)	0.19 (0.06–0.61)	0.19 (0.06–0.61)	38.7 (5.0–296.9)
C. Two sample subsets defined by the RCC-gene set in Fig. 1c (p = 0.003)					
Group A, n = 35	0.94 (0.86–1.00)	0.90 (0.80–1.00)	0.90 (0.80–1.00)	0.90 (0.80–1.00)	Reference group
Group B, n = 25	0.71 (0.55–0.92)	0.56 (0.38–0.81)	0.46 (0.27–0.78)	0.46 (0.27–0.78)	5.4 (1.5–19.3)
D. Two sample subsets defined by the combined gene set in Fig. 1d (p = 0.029)					
Group A, n = 14	NR	NR	NR	NR	Reference Group
Group B, n = 46	0.80 (0.69–0.93)	0.66 (0.52–0.84)	0.59 (0.43–0.82)	0.59 (0.43–0.82)	NE
E. Three sample subsets defined by the combined gene set in Fig. 1e (p < 0.001)					
Group A, n = 14	NR	NR	NR	NR	Reference Group
Group B1, n = 17	NR	NR	NR	NR	NE
Group B2, n = 29	0.68 (0.53–0.88)	0.47 (0.29–0.74)	0.39 (0.22–0.70)	0.39 (0.22–0.70)	NE
F. Two sample subsets defined by the combined gene set in Fig. 1F (p < 0.001).					
Good group, n = 32	NR	NR	NR	NR	Reference Group
Poor group, n = 28	0.67 (0.52–0.87)	0.48 (0.32–0.74)	0.40 (0.23–0.70)	0.40 (0.23–0.70)	NE

*NR* no recurrence, *HR* hazard rario, *NE* hazard ratio can not be estimated when one group has no events

### Analysis of GIST samples using the combined gene set

**Fig. 2:**
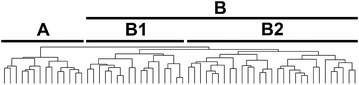
Clustering of gene expression in the 60 GIST samples. The samples were clustered using the probes in the pooled gene set as described in the text. *Groups A* and *B* (= B1 + B2) are defined by the *first branch point* in the clustering. *Groups B1* and *B2* are defined by the *second branch point* in *Group B*

Hierarchical clustering of the GIST samples was also performed using the combined gene set (AF-gene set, OVCA-gene set, and RCC-gene set) (Fig. 1d). Kaplan–Meier analysis was performed using the two sample sets defined by the first branch point. The probability of not developing a metastasis by 6 years was 0.59 for Group B, while none recurred in Group A (Fig. 1d; Table 1D, p = 0.029). In Group B, clustering was evident between two subgroups of sufficient sample size to analyze independently (Fig. 2). The probability of not developing a recurrence by 6 years was 0.39 for Group B2, while none of the patients in Group A or Group B1 recurred (Fig. 1e; Table 1E, p < 0.001 for comparisons between each of the 3 sets). We also grouped the samples into 2 groups defined as “good prognosis” or “poor prognosis”. Samples were defined as “good” prognosis if they were in Group A in the clustering by at least 2 of the 3 gene sets (AF-, OVCA-, or RCC; n = 30). The probability of not developing a metastasis by 6 years was 0.40 for the “poor” prognosis group, while none of the patients in the “good” prognosis group recurred (Fig. 1f; Table 1F, p < 0.001).

### Effect of Miettinen risk score on probability of developing recurrence

**Fig. 3:**
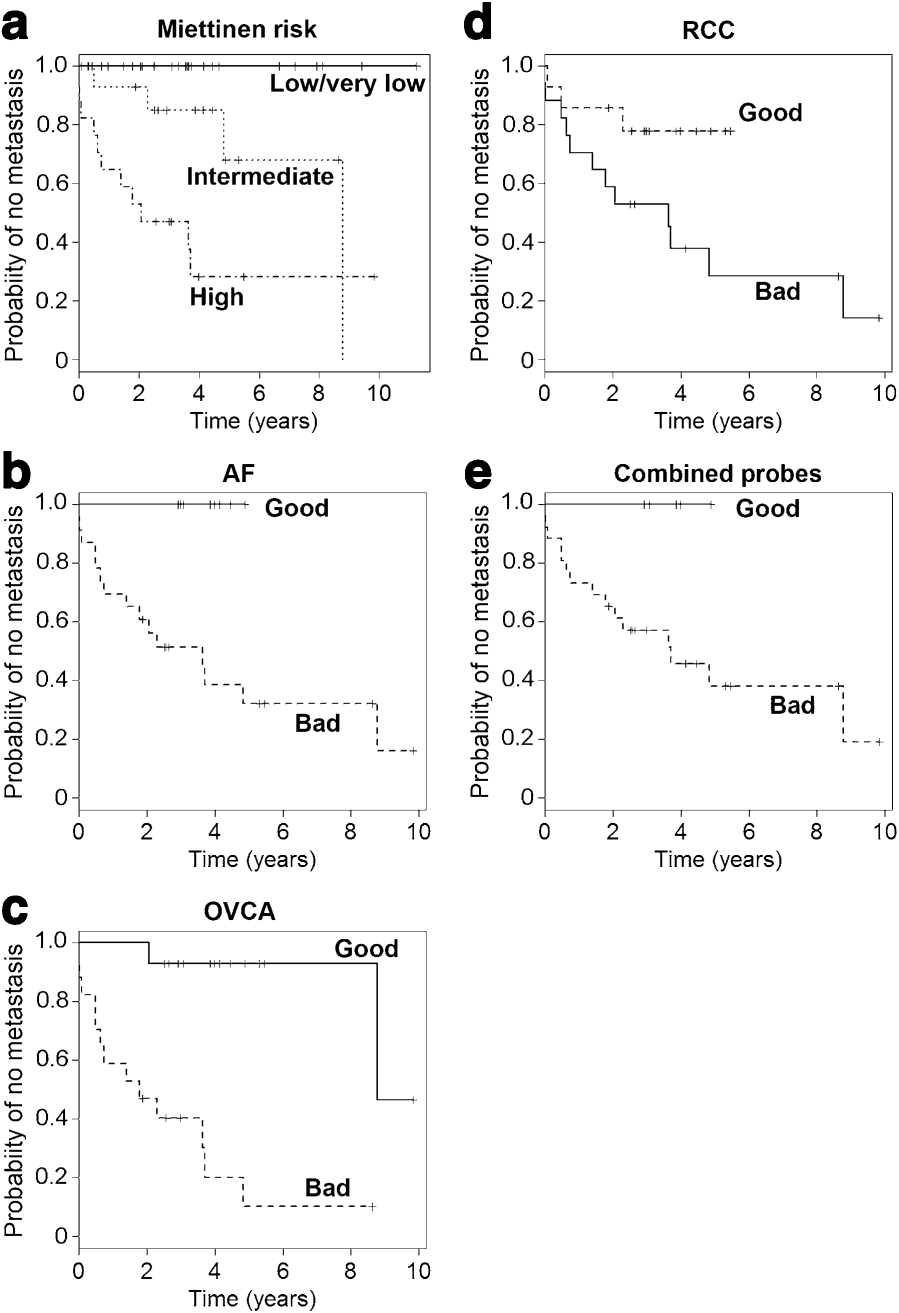
Kaplan–Meier analysis of the time to development of metastases. Kaplan–Meier analysis of the time to development of metastases for Miettinen risk groups of all GIST samples (**a**). Miettinen risk identified distinct risk groups (p < 0.001), *panel* **a**. When the 31 high- and intermediate-risk GIST samples from *panel* **a** were examined for their grouping using the individual AF-gene set (**b**), OVCA-gene set (**c**), RCC-gene set (**d**), and the combined gene set (**e**) in Fig. 1, the time to development of metastasis differed between *groups A* and *B* (**b**, p < 0.001; **c**, p = 0.003; **d**, p = 0.029; and **e**, p = 0.029)

As some prognostic criteria correlate with recurrence of GIST, we questioned whether this scoring method might be improved by combining it with our clustering patterns. When the GIST samples were analyzed according to Miettinen risk status [11], none of the 29 patients in the low or very low risk groups recurred, yet 16 of 31 patients who scored in the high- or intermediate-risk score by Miettinen risk also did not recur (Fig. 3a; Table 2A).

**Table 2:** Probability (95 % CI) of no metastasis as a function of time

Time (years)	2	4	6	8	HR
A. Four sample subsets defined by Miettinen risk score (Fig. 3a, p < 0.001)					
High risk, n = 17	0.53 (0.34–0.83)	0.28 (0.12–0.68)	0.28 (0.12–0.68)	0.28 (0.12–0.68)	3.4 (1.1, 10.9)
Intermediate risk, n = 14	0.93 (0.80–1.00)	0.85 (0.68–1.00)	0.68 (0.42–1.00)	0.68 (0.42–1.00)	Reference group
Low risk, n = 16	NR	NR	NR	No follow-up	Not estimable
Very low risk, n = 13	NR	NR	NR	NR	Not estimable
B. Two sample subsets among the high- and intermediate-risk samples defined by the AF-gene set in Fig. 1a (Fig. 3b, p = 0.01)					
Good group, n = 8	NR	NR	No follow-up	No follow-up	Reference group
Bad group, n = 23	0.61 (0.44–0.85)	0.39 (0.22–0.68)	0.32 (0.17–0.63)	0.32 (0.17–0.63)	Not estimable
C. Two sample subsets among the high- and intermediate-risk samples defined by the OVCA-gene set in Fig. 1B (Fig. 3C, p < 0.001)					
Good group, n = 14	NR	0.93 (0.80–1.00)	0.93 (0.80–1.00)	0.93 (0.80–1.00)	Reference group
Bad group, n = 17	0.47 (0.28-0.78)	0.20 (0.06–0.63)	0.10 (0.02–0.61)	0.10 (0.02–0.61)	19.9 (2.6–154.0)
D. Two sample subsets among the high- and intermediate-risk samples defined by the RCC-gene set in Fig. 1c (Fig. 3d, p = 0.045)					
Good group, n = 17	0.59 (0.40–0.88)	0.38 (0.20–0.72)	0.28 (0.12–0.67)	0.28 (0.12–0.67)	3.4 (1.0, 12.3)
Bad group, n = 14	0.86 (0.69–1.00)	0.78 (0.59–1.00)	No follow-up	No follow-up	Reference group
E. Two sample subsets among the high- and intermediate-risk samples defined by the combined gene set in Fig. 1d (Fig. 3e, p = 0.06129).					
Good group, n = 5	NR	NR	No follow-up	No follow-up	Reference group
Bad group, n = 26	0.65 (0.49–0.87)	0.46 (0.29–0.72)	0.38 (0.21–0.68)	0.38 (0.21–0.68)	Not estimable

*NR* no recurrence, *HR* hazard ratio, *NE* hazard ratio can not be estimated when one group has no events

We went back to the hierarchical clustering performed with each of the gene sets in Fig. 1 to determine where these 31 patients with high- and intermediate-risk from the Miettinen score had been grouped, i.e. were they in Group A (good prognosis) or Group B (bad prognosis) (Fig. 1a–d). The probability of no metastasis for these 31 patients is shown in Fig. 3b–e and Table 2. Of interest is the finding that many of the 31 patients who were classified as high- or intermediate-risk by the Miettinen score were grouped as good prognosis using our gene sets and did not recur. The rate of recurrence in the “good risk” group was 0 % (0/8) for the AF-gene set, 14 % (2/14) for the OVCA-gene set, 18 % (3/17) for the RCC-gene set, and 0 % (0/5) for the pooled-gene set. Interestingly, among the “good” groups of high- and intermediate-risk samples defined by the AF-gene set, 0/3 high-risk and 0/5 intermediate-risk tumors recurred; 1/5 high- and 1/9 intermediate-risk tumors recurred in the “good” group identified by clustering with the OVCA-gene set, and 2/7 high- and 1/7 intermediate-risk tumors recurred when clustered with the RCC-gene set. Among the “bad” prognosis group defined by clustering with the AF-, OVCA-, and RCC- gene sets, 11/14 high- and 4/9 intermediate-risk, 10/12 high- and 3/5 intermediate-risk, and 9/10 high- and 3/7 intermediate-risk samples recurred, respectively.

## Discussion

The biologic heterogeneity of GISTs, as with other soft tissue sarcomas, introduces complexities in deciding optimal treatment. This study used hierarchical clustering with gene sets derived from earlier studies of various biochemical pathways in aggressive fibromatosis, renal cell carcinoma, and ovarian carcinoma [36–39, 43] to examine 60 GIST samples using Agilent chip expression profiling. The analyses separated the GIST samples into at least two groups with different probabilities of developing metastatic disease. Although the gene sets were derived using biochemical pathways, we did not observe simple differences in biochemical pathways between the groups; possibly with a larger sample set, more detailed biochemical differences will become evident. Our data suggest that appreciation of these GIST subsets with distinct clinical behavior could be used to stratify GIST patients in clinical trials and in patient management. Miettinen risk group classification also identified distinct risk groups in our 60 GIST cases. In particular, our analysis also identified subsets of Miettinen high- and intermediate-risk samples that different in the risk of metastasis. When the high- and intermediate-risk GIST samples were examined without the low- and very low-risk samples, the individual AF-gene set, OVCA-gene set, RCC-gene set, and the combined gene set were associated with the time to development of metastasis. This finding suggests that further characterization of recurrence risk among samples classified as high- or intermediate-risk is possible. Furthermore, these results validate the potential role of the use of these gene sets in predicting the behavior of heterogeneous tumor sets.

These gene sets have also been shown to separate sets of soft tissue sarcoma samples into groups with different metastatic behavior [25, 26]. A gene set of 67 genes involved in mitosis and control of chromosome integrity, termed the complexity index in sarcomas (CINSARC), also predicts metastasis outcome in non-translocation dependent soft tissue sarcomas [23]. Both the gene sets used here and the CINSARC [23, 33] gene set identified subsets of the GIST samples that differed in time to recurrence. These data support the potential use of these gene sets to predict biological behavior in GIST as well as other soft tissue sarcomas. Only 11 of the 67 genes in the CINSARC gene set were also present in our pooled gene set.

Other methods of examining genetic heterogeneity may also be helpful. A recent study found that chromosomal changes detected by comparative genomic hybridization (CGH) were predictive of GIST outcome [33]. This study, as well as a second study, also found that a “genomic index” calculated from the number of chromosomal alterations (segmental gains and losses), and number of chromosomes involved was a strong predictor of recurrence as well [33, 44]. Another study using array-based analysis of gene copy number separated 42 GISTs into 4 groups with different survival rates [35].

## Conclusions

Gene expression profiles may provide a useful technique to better predict long-term outcomes after surgery in patients with GIST and other sarcomas. Such information could be used to restrict the use of adjuvant therapy and reduce heterogeneity among groups in clinical trials. Due to the limited sample size of our study, we examined the identification of only two subsets of the GIST sample set with different metastatic propensity. The ability to detect multiple subgroups is highly dependent on the number of samples and the distribution of samples among the various groups. With larger sample sets, it may be possible to further refine classification and identify clinically useful heterogeneity. In addition, although gene expression analysis may provide a useful indicator of long-term outcomes, it should be used in combination with standard prognostic factors in order to have maximum predictive value [8]. For example, in this study, further characterization of recurrence risk among samples classified as high-or intermediate-risk was possible. These results also validate the potential role of the use of these gene sets in predicting the behavior of heterogeneous tumor sets. Several different gene sets appear to separate the samples into 2 groups with different behavior.

## Additional file

10.1186/s12967-016-0802-3 Clustering of gene expression in the 60 GIST samples. The samples were clustered using the probes in the indicated gene set as described in the text. Panel A, all probes; panel B, AF-gene set; panel C, OVCA-gene set; panel D, RCC-gene set.

## Abbreviations

GIST: gastrointestinal stromal tumor
RCC: clear cell renal cell carcinoma
OVCA: ovarian cancer
AF: aggressive fibromatosis
