# Supplementary figures and images for: Gene expression identifies heterogeneity of metastatic behavior among gastrointestinal stromal tumors
Source: J Transl Med. 2016 Feb 13;14:51. doi: 10.1186/s12967-016-0802-3 (PMC4752787; doi:10.1186/s12967-016-0802-3)

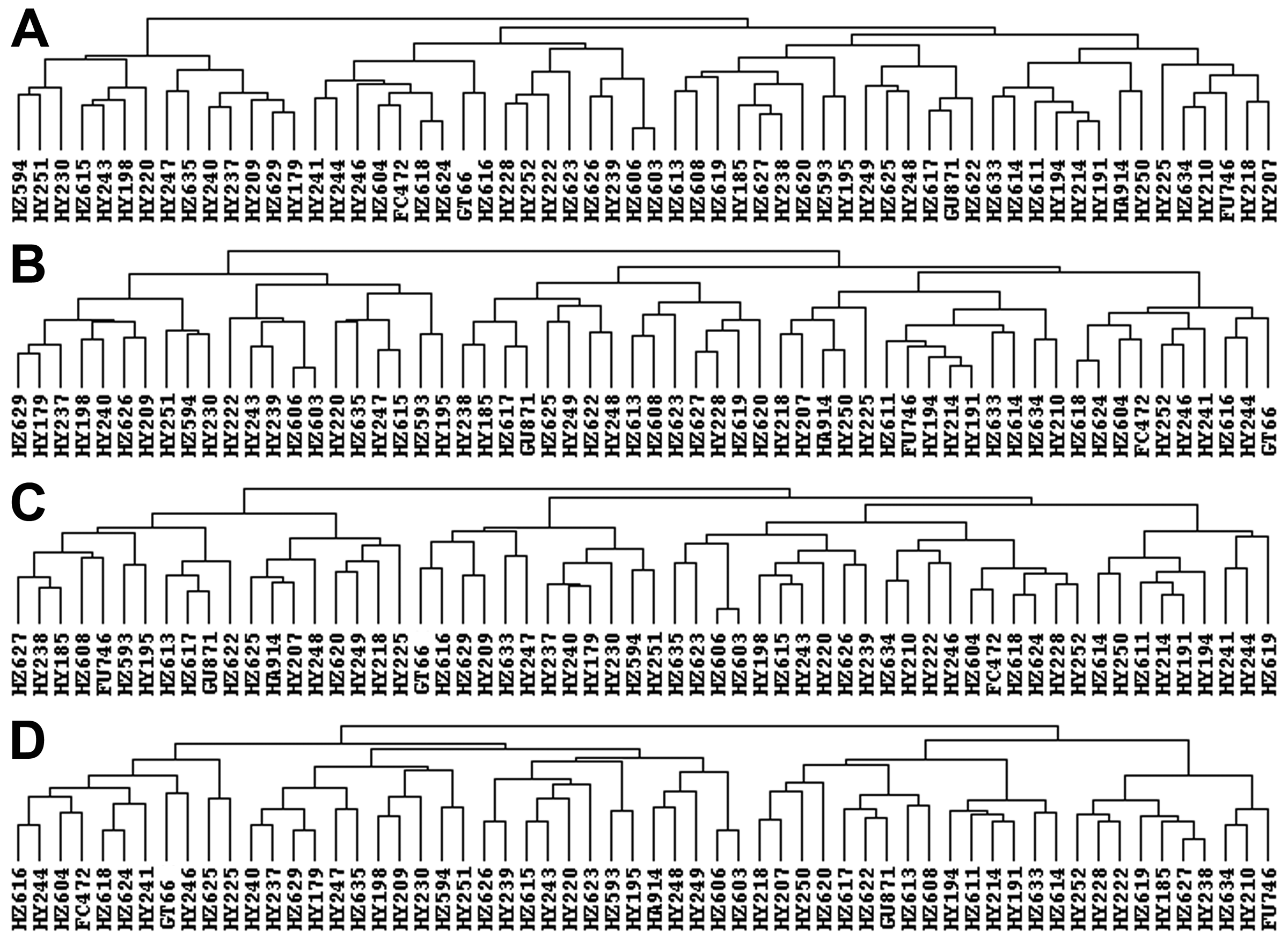

Supplement: Supplementary file 1 — 10.1186/s12967-016-0802-3 Clustering of gene expression in the 60 GIST samples. The samples were clustered using the probes in the indicated gene set as described in the text. Panel A, all probes; panel B, AF-gene set; panel C, OVCA-gene set; panel D, RCC-gene set. [file 12967_2016_802_MOESM1_ESM.tif]
